# A case of pulmonary tuberculosis with hemoptysis from a peripheral pulmonary aneurysm

**DOI:** 10.17179/excli2021-4279

**Published:** 2021-10-04

**Authors:** Kodai Furuyama, Noriyuki Hirama, Shigeyuki Fukushima, Minoru Inage, Hiroki Ota, Kento Sato, Keiko Yamauchi, Masamichi Sato, Akira Igarashi, Sumito Inoue, Masafumi Watanabe

**Affiliations:** 1 Respiratory Medicine, Okitama Public General Hospital, 2000, Nishi-Otsuka, Kawanishi, Yamagata, Japan; 2 Department of Cardiology, Pulmonology, and Nephrology, Yamagata University Faculty of Medicine, 2-2-2 Iida-Nishi, Yamagata 990-9585, Japan

**Keywords:** pulmonary tuberculosis, pulmonary aneurysm, hemoptysis

## Abstract

An 88-year-old woman visited our hospital for hemoptysis due to ruptured peripheral pulmonary aneurysm diagnosed by contrast computed tomography (CT) and angiography. Her bleeding was stopped by interventional radiology vascular embolization. She was diagnosed with pulmonary tuberculosis due to a positive acid-fast bacillus (AFB) smear test following admission and the positive polymerase chain reaction for tuberculosis, despite no obvious cavity lesions or scatter shadows on CT. The causes of hemoptysis due to pulmonary tuberculosis are known to be Rasmussen aneurysm, in which the blood vessel wall adjacent to the lung cavity is thinned to form an aneurysm, or bleeding from the bronchial artery. In this case, it was considered that the inflammation caused by pulmonary tuberculosis spread directly to the pulmonary artery and formed a pulmonary aneurysm without forming a cavity. Similar cases have been rarely reported. Clinicians need to consider pulmonary tuberculosis as the cause of pulmonary aneurysm, even without cavity lesions in the lungs. It is important to perform AFB examination to diagnose pulmonary tuberculosis.

## Introduction

Although the number of patients with pulmonary tuberculosis (TB) is decreasing in Japan, the rate of decline in Japan shows that pulmonary TB is still not considered as an unusual condition here. Especially among the elderly, there is a possibility of relapse, after infection at a young age, due to aging. Imaging findings of pulmonary TB are characterized by cavities and scattered shadows, but it is difficult to diagnose based on imaging findings alone because atypical cases of findings exist. Hemoptysis is one of the characteristic findings of pulmonary TB.

The common courses of hemoptysis in pulmonary TB include endobronchial TB, tubercular cavity, bronchiectasis, and rarely vascular aneurysms (Gupta et al., 2019[2]). Probably the most well-known aneurysm associated with pulmonary TB is the Rasmussen aneurysm, which forms an aneurysm by thinning the blood vessel wall adjacent to the cavity of the lung, and it is combined with 4 %-5 % cases of chronic pulmonary TB (Theodoropoulos et al., 2013[6]). In this case, we report a case of hemoptysis without the cavity findings characteristic of pulmonary TB.

## Case Report

An 88-year-old woman visited our emergency department due to hemoptysis. She had hypertension, diabetes mellitus, and dementia due to cerebral infarction. She also had interstitial pneumonia and had been followed up at our hospital but had voluntarily interrupted treatment.

**Figure 1:**
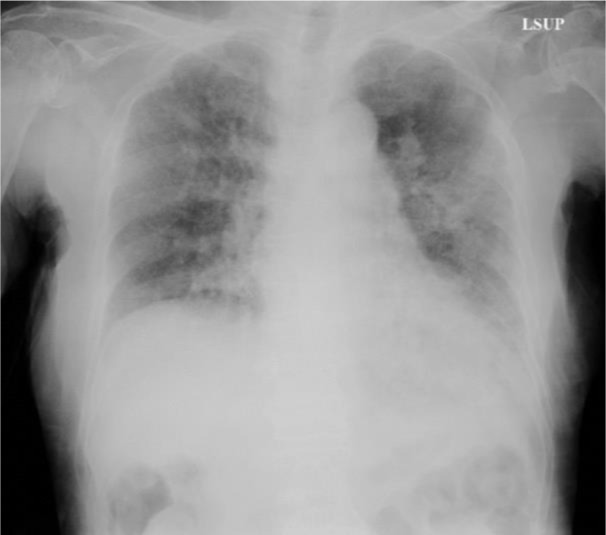
Chest radiography on admission showed bilateral reticular shadows but no finding of cavity lesions.

**Figure 2:**
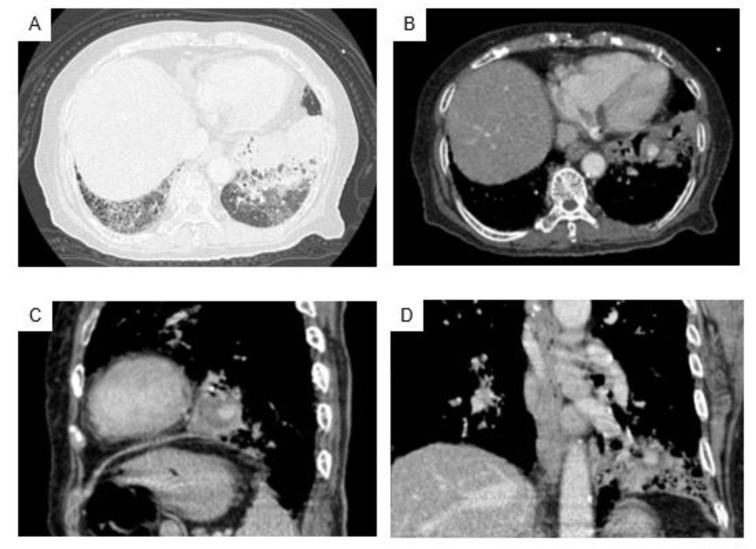
Contrast-enhanced chest CT on admission revealed grand-grass shadow and findings of honeycomb lung in bilateral lower lungs, and infiltrating shadow in the lower left lung field (A). An aneurysm with a contrast effect was found inside the infiltrated shadow in the left lower lung, without any obvious cavity lesion (B: axial view, C: sagittal view, D: coronal view).

At the time of this admission, her vital signs were normal, and fine crackles were heard from bilateral lower lungs. White blood cells count was increased (19,140 /µL) and serum level of C-reactive protein was slightly increased (3.71 mg/dL). Her hemoglobin level was 8.0 g/dl, hematocrit was 23.1 %, Mean Corpuscular Volume was 80.5 fL, platelet count was 290,000 /µL. Antinuclear antibody, perinuclear-antineutrophil cytoplasmic antibody (ANCA) and C-ANCA were all negative. The result of Interferon-Gamma Release Assay was indeterminate. Chest radiography (Figure 1(Fig. 1)) showed bilateral reticular shadows but no finding of cavity lesions. Contrast-enhanced chest CT showed a honeycomb lung in the right lower lung field and infiltrative shadow and ground glass shadow in the left lower lung field (Figure 2A(Fig. 2)). A contrast-enhancing aneurysm was seen within the infiltrative shadow in the left lower lung, but no obvious cavitary lesion was seen (Figure 2B-D(Fig. 2)).

**Figure 3:**
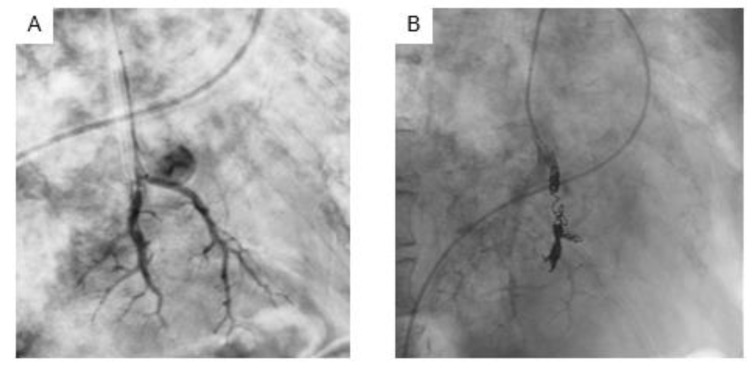
Angiography of left pulmonary artery showed aneurysm in pulmonary artery (A). Endovascular coil embolization for the aneurysm in pulmonary artery was successfully performed (B).

We thought the patient's hemoptysis was caused by an aneurysm in the lower left lung field. We performed angiography (Figure 3(Fig. 3)) and successfully performed endovascular coil embolization of an aneurysm in the pulmonary artery. After these treatments, sputum screening tested positive for acid-fast bacillus. She was diagnosed with pulmonary TB based on the result of polymerase chain reaction positive for mycobacterium TB. She was treated with isoniazid (INH), rifampicin (RFP), and ethambutol (EB) without pyrazinamide (PZA) due to problems with liver function. No recurrence of hemoptysis was observed after successfully treating pulmonary TB.

## Discussion

Hemoptysis in patients with pulmonary TB is one of its major symptoms. Direct vascular involvement by pulmonary TB may also occur, as both arteries and veins may demonstrate an active mycobacterial-induced necrosing granulomatous vasculitis, resulting in inflammation with aneurysmal changes, broncho-pulmonary, and arterio-venous connections (Seedat and Seedat, 2018[5]). In some cases (up to 5 % in patients with cavitary TB disease), hemoptysis is related to the Rasmussen aneurysm (Seedat and Seedat, 2018[5]; Santelli et al., 1994[4]), thought to be caused by the weakening of the pulmonary artery wall from adjacent to cavity lesion of pulmonary TB. The arterial wall gradually weakens as the granulation tissue replaces both the adventitia and the media. It gradually replaces fibrin, thinning the arterial wall, forming a pseudoaneurysm, and then rupturing with bleeding (Auerbach, 1939[1]; Yanase et al., 1994[7]).

In this case, CT showed no findings of cavities due to pulmonary TB. Angiography showed that the responsible vessel was the pulmonary artery rather than the bronchial artery. Initially, we did not suspect pulmonary TB from CT findings, but we were able to diagnose pulmonary TB by screening sputum culture examination. Some cases have been reported in which pulmonary TB formed an aneurysm in the pulmonary artery without forming a cavity and caused hemoptysis (Yanase et al., 1994[7]). It is speculated that inflammation from pulmonary TB directly affects the pulmonary arteries and causes the hemoptysis. Yanase et al. reported that postmortem pathological findings show that a tuberculous lesion in the lung destroys the upper left trunk bronchial wall, and inflammation spreads to the pulmonary artery, causing thinning of the pulmonary artery and forming an aneurysm in the pulmonary artery, protruding into the bronchial lumen. They describe that the aneurysm in the pulmonary artery was not formed in a cavity lesion in the lung like the Rasmussen aneurysm, as no cavity lesion was found in the lungs in this case (Yanase et al., 1994[7]). In another case report, long-term bronchial inflammation is thought to have spread to the pulmonary artery in patients with bronchiectasis (Nishimura et al., 2021[3]). Also in this case, it is thought that long-term inflammation might have spread to the pulmonary artery and formed an aneurysm in pulmonary artery.

However, we have not yet confirmed a detailed mechanism for hemoptysis without cavity lesions in the lung. We must collect similar cases to elucidate the mechanism of hemoptysis. In conclusion, we must consider that pulmonary TB might cause hemoptysis due to an aneurysm of pulmonary artery, even without the cavity formation that is typical of pulmonary TB. Early detection and diagnosis of pulmonary TB are very important because delaying the diagnosis of pulmonary TB increases the risk of infection to medical personnel and to other patients.

## Acknowledgements

The authors would like to thank Enago (www.enago.jp) for English language review.

## Conflict of interest

The authors declare that they have no conflict of interest.
